# Artificial Intelligence Is Reshaping Healthcare amid COVID-19: A Review in the Context of Diagnosis & Prognosis

**DOI:** 10.3390/diagnostics11091604

**Published:** 2021-09-02

**Authors:** Rajnandini Saha, Satyabrata Aich, Sushanta Tripathy, Hee-Cheol Kim

**Affiliations:** 1 School of Biotechnology, KIIT Deemed to be University, Bhubaneswar 751024, Odisha, India; saharajnandini@gmail.com; 2 Wellmatix Corporation Limited, Changwon 51395, Korea; satyabrataaich@gmail.com; 3 School of Mechanical Engineering, KIIT Deemed to be University, Bhubaneswar 751024, Odisha, India; 4 Institute of Digital Anti-Aging Healthcare, College of AI Convergence, u-AHRC, Inje University, Gimhae 50834, Korea

**Keywords:** AI, COVID-19, diagnosis, interpretive structural modeling, healthcare

## Abstract

Preventing respiratory failure is crucial in a large proportion of COVID-19 patients infected with SARS-CoV-2 virus pneumonia termed as Novel Coronavirus Pneumonia (NCP). Rapid diagnosis and detection of high-risk patients for effective interventions have been shown to be troublesome. Using a large, computed tomography (CT) database, we developed an artificial intelligence (AI) parameter to diagnose NCP and distinguish it from other kinds of pneumonia and traditional controls. The literature was studied and analyzed from diverse assets which include Scopus, Nature medicine, IEEE, Google scholar, Wiley Library, and PubMed. The search terms used were ‘COVID-19’, ‘AI’, ‘diagnosis’, and ‘prognosis’. To strengthen the overall performance of AI in COVID-19 diagnosis and prognosis, we segregated several components to perceive threats and opportunities, as well as their inter-dependencies that affect the healthcare sector. This paper seeks to pick out the crucial fulfillment of factors for AI with inside the healthcare sector in the Indian context. Using critical literature review and experts’ opinion, a total of 11 factors affecting COVID-19 diagnosis and prognosis were detected, and we eventually used an interpretive structural model (ISM) to build a framework of interrelationships among the identified factors. Finally, the matrice d’impacts croisés multiplication appliquée á un classment (MICMAC) analysis resulted the driving and dependence powers of these identified factors. Our analysis will help healthcare stakeholders to realize the requirements for successful implementation of AI.

## 1. Introduction

This manuscript emphasizes potential areas of academic studies that are possible to be impacted via COVID-19. The aim of this paper is to develop diagnostic and prognostic models via Computed Tomography to deal with COVID-19 patients tormented by pneumonia and to come to be privy to the research regions associated with COVID-19 diagnosis and prognosis. It may help enhance the information of this disorder and describe the psychological effects of this pandemic and how these could trade as the ailment spreads.

The novel coronavirus, specifically SARS-CoV-2, emerged in December 2019 to cause a respiratory sickness known as COVID-19, which has shown to be a difficult illness with severity levels ranging from moderate to severe, as well as the risk of organ failure and death. Around 213 countries and territories have been affected with a hard dependency on 386,600 deaths worldwide [1,2]. COVID-19 has triggered a global health catastrophe that has had a profound impact on how we perceive our global and everyday lives. Not only does the cost of contagion and the different types of transmission endanger our sense of entity, but the protection safeguards to cease the virus from spreading also necessitate social distancing by refraining from doing what is inherently human, which is to seek solace in the ventures of others. According to the World Health Organization (WHO), the most common result of elevated COVID-19 is severe pneumonia [3]. COVID-19 can be fatal for people who develop symptoms in their lungs [4,5]. Doctors use imaging studies to search for swelling, inflammation, or fluid inside the lungs to diagnose pneumonia. X-ray or CT (computed tomography) scans is recommended to identify pneumonia [3,6].

AI plays a key role in drug repurposing. Bioinformatics is a powerful tool for accelerating drug discovery. Given the current circumstances, applying bioinformatics methods to solve the problem is critical [7]. AI approaches are increasingly being used in drug discovery to solve previously tough problems such as predicting characteristics, creating compounds, and optimizing synthetic routes. Tools such as binding simulations, protein modeling, and computational chemistry are commonly employed in related fields; nevertheless, they should be used with caution because the conformational space is complicated and entropic contributions from the surrounding solvent are significant. Various types of AI are utilized to create compounds and models, which are then used for training and supervised learning [8].

**Figure 1:**
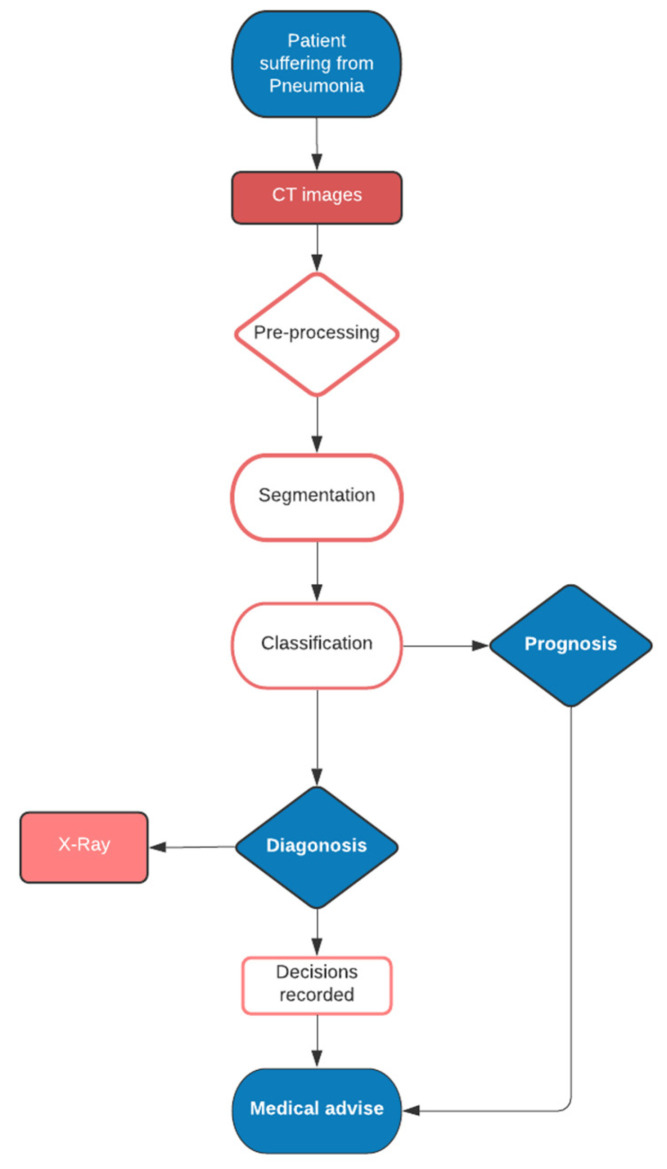
Diagnostic and prognostic management.

AI is being used to help fight the viral pandemic that has overtaken the entire world since the year 2020 began. The press and scientific community are echoing the high hopes that data science and AI may be utilized to combat the coronavirus and “fill in the blanks” left by science [9,10,11]. AI technology enables radiologists and physicians make faster diagnosis. Importantly, our AI tool revealed crucial scientific markers that were linked to the features of the Novel Coronavirus Pneumonia (NCP) disease. Our AI machine was able to deliver accurate medical prognosis using medical data, allowing physicians to focus on proper early medical control and spend resources correctly. Outbreaks result in a significant increase in the number of people seeking medical help. To lessen the strain on the health-care system, high-quality diagnosis and prognosis metrics have been implemented. Prediction models, which use a few predictors (variables or features) to assess the probability of infection or poor infection outcomes, may help the scientific team screen patients while allocating limited healthcare resources. Prediction models have already been developed and worked on, starting with rule-based scoring systems and progressing to deep learning; however, the results are not rewarding enough [10,12]. A layout of diagnostic and prognostic management in the context of the AI-based healthcare sector is shown in Figure 1.

**Figure 2:**
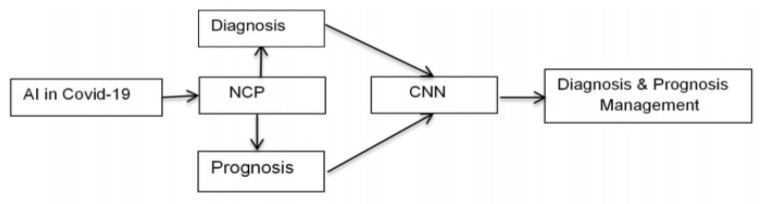
Framework of the paper.

The framework for the application of AI in diagnosis and prognosis is shown in Figure 2. This paper is structured as follows: Section 2 entails the significance of diagnosis and prognosis and how AI can tame the diagnosis and prognosis through CNN procedure. Section 3 focuses on the proposed models to estimate the clinical diagnosis and prognosis. Section 4 highlights the results and discussion. Section 5 discusses the managerial implications. Section 6 discusses the practical implications. Section 7 delves into the findings based on the AI in COVID-19 diagnosis and prognosis.

## 2. Related Work

This section is classified into four subsections. The first subsection presents the factors related to COVID-19 diagnosis and prognosis. The second part focuses the application of AI in healthcare and the third section entails the role of AI in diagnosis and prognosis. Finally, subsection four implies the significance of statistical analysis of AI in healthcare.

### 2.1. Factors Related to COVID-19 Diagnosis and Prognosis

AI refers to the simulation of human intelligence in computers that are trained to think and act like humans. The phrase can also refer to any computer that has features akin to a human mind, such as learning and problem-solving abilities. An advanced technology that is helpful to tackle the COVID-19 pandemic is AI. This technology helps to screen, monitor, and forecast patients today and in the future. Early detection and diagnosis of infection are the main applications of this AI. AI is used for medication and vaccine production and workload reduction in health staff [6,13]. A Computerized Tomography (CT) scan combines a series of X-ray images taken from different angles across the body to create cross-sectional images (slices) of your bones, blood vessels, and soft tissues. X-rays are less informative than CT scan images. Imaging may help to determine the severity of the disease in patients with extreme symptoms. CT scans or X-rays can help assess a patient care plan by using laboratory testing, detailed medical history, and a physical examination [2,10,14].

NCP (Novel Coronavirus Pneumonia) is an inflammatory lung infection in small airbags. It makes it difficult to breathe because their lungs gets filled with so much fluid and pus. Significant shortness of breath, cough, fever, chest pain, chills, or weariness can be present. The majority of people with COVID-19 have moderate to mild symptoms such as cough, fever, and shortness of respiration. However, some people who capture new coronaviruses develop extreme pneumonia. The pneumonia of COVID-19 is a dangerous and deadly disease [3,15].

Medical advice suggests a positive test result can only be reported nearly a week after exposure to COVID-19. Evidence shows that the experiment is generally less effective within the three days of exposure, and the safest time to get checked is five to seven days after you have been exposed. The advice is also to put the used mask fabric in a closed bin and wash your hands immediately [13,16]. Contract data COVID-19 lists a set of complications for ongoing clinical trials, including a higher probability of missing outcome details. Trial experts should examine the management plans for missing facts and statistical analysis, according to international standards on clinical studies, although there are no clear suggestions. [10,17,18]. Physical examination, pneumonia is the most common severe type of COVID-19 after the initial surgery. In these conditions, fever, cough, dyspnea, and chest imaging anomalies are recurrent [11,19]. The swift consultation by experts responds to the request made in connection with a temperature, humidity, and possible seasonal reduction and re-emergence of the SARS-CoV2 virus by the Office of the Science and Technology Pacific (OSTP) [20,21].

In older people, the risk of serious COVID-19 disease is increased. In their 50s, for instance, people are more at risk for serious illness than in their 40s. Similarly, in general, persons in their 60s or 70s are more vulnerable than persons in their 50s to serious disease [11,22]. Regarding gender, evidence from previous epidemics, such as the outbreak in 2002–2003 of SARS coronavirus, suggests that both men and women are vulnerable to the virus as well as to the infection due to gender and sexual factors [16,23]. When it comes to body temperature, regardless of the outside temperature or weather, the average human body temperature remains between 36.5 °C and 37 °C. Temperature screening alone is not an efficient means to avoid the spread globally, as the infected person may be in the incubation phase, may not express noticeable symptoms early in the course of the disease, and may dissimulate fever using antipyretic agents. To ensure that a proper risk assessment and future contact monitoring for incoming travelers is feasible, it is better to provide prevention advice to passengers and to obtain health declarations at arrival with contact information [15,23].

There is growing uunderlying disease evidence for the increased risk of death due to COVID-19 in people with established chronic conditions or affected immune systems due to disability. Elderly people and those with underlying medical disorders such as cardiovascular disease, diabetes, chronic respiratory illness, and cancer are more likely to develop the disease [17,24].

### 2.2. Past Studies Regarding the Application of AI in Healthcare

The objective of AI is to emulate cognitive abilities. The increasing quality of healthcare data, combined with the rapid advancement of analysis methodologies, is causing a paradigm shift in healthcare. AI may be used on a variety of healthcare data sets (structured and unstructured). Popular AI methodologies include structured data machine learning methods such as the classic support vector machine and neural network, as well as unstructured data deep learning and natural language processing [25,26]. Cancer, neurology, and cardiology are three major illness areas that use AI techniques. In certain areas of healthcare, professionals can use AI to help them make better healthcare decisions, and it may even be able to replace human judgments (e.g., radiology). The rapid development of big data analytic methodologies, as well as the rising availability of healthcare data, has made recent effective uses of AI in healthcare possible. When used in conjunction with the right clinical questions, substantial results can be achieved systems can reveal therapeutically important information hidden in large amounts of data, allowing clinicians to make better decisions [9,13,27].

CNN (Convolutional Neural Network) is a deep neural network type, most frequently used for the analysis of visual imagery. Nanoparticles and deep neural networks are used to detect viruses [20,28]. Radiologists employ medical imaging to diagnose and treat illnesses. Chest X-rays are now commonly acknowledged as the first-line imaging technology, with chest CT reserved for the most severely ill patients or when chest X-rays and clinical presentation are inconclusive [6,29].

AI systems must be “trained” utilizing data generated by clinical activities such as screening, diagnosis, and therapy assignment in order to understand similar groups of subjects, relationships between subject features, and desired outcomes. Demographics, medical notes, electronic records from medical equipment, physical examinations, clinical laboratory tests, and photographs are just some of the types of clinical data [30].

Physical examination reports and clinical laboratory findings are the other two key data sources. They differ from imaging, genetic, and electrocardiography (EP) data in that they contain a large amount of unstructured narrative content, such as clinical notes, that is difficult to interpret [16,31]. As a result, AI applications focused on converting unstructured data into machine-readable electronic medical records (EMR).

There are two types of AI devices available. The first category includes machine learning (ML) approaches for analyzing structured data such as imaging, genomics, and EP data. Machine learning algorithms are used in medical applications to classify people’s characteristics and predict disease consequences. Natural language processing (NLP) technologies, on the other hand, extract information from unstructured data sources like clinical notes and medical journals to supplement and augment organized medical data. Machine learning techniques are used to convert texts into machine-readable structured data that can subsequently be studied [32,33].

### 2.3. AI in Diagnosis and Prognosis

The diagnosis is the medical name for the patient’s illness based on its symptoms. The initial step in the diagnostic process is to gather information about the patient’s medical history. The patient is next subjected to a scientific evaluation. Prognosis is a term used to describe the methodical assessment of an illness’s anticipated progression and outcome. The study is based on statistics from the average course of an effective disease, the patient’s physical and mental state, any associated diseases (if any), the prescribed medications, and case-specific circumstances. The full analysis covers the expected duration, outcome, and outline of the disease’s spread [9,11,34].

The maximum probable disease that is in all likelihood to occur through COVID-19 is pneumonia. Many COVID-19 sufferers infected by using the SARS-Cov-2 virus increase pneumonia and swiftly develop respiratory failure. Eventually, they may increase multiorgan failure [35]. A COVID-19 prognosis is confirmed via an advantageous molecular PCR (polymerase chain reaction) check. CT (computed tomography) [13,36] has to function as a crucial tool in the analysis process. It has the fastest turnaround time than a molecular diagnostic test and offers more specified info related to pathology. It proves to be a higher qualitative measurement of the lesion (vicinity of the lung tissue) and the seriousness of the lung volume inclusion may furthermore have prognostic involvement [10,14,37].

By generating an AI parameter utilizing both clinical data and CT characteristics, an accurate clinical prognostic model is established, allowing physicians to devise strategies for early tracking and care of these patients. The COVID-19 diagnostic parameter is a combination of lung lesion segmentation and diagnosis analysis [38]. The respiratory organ lesions taken are manually annotated identification is confirmed by PCR check and quantified respiratory organ lesions are confirmed via CT scans. To recognize the patterns of interstitial lung disease (ILD), the CNN (Convolutional neural network) model came into action [39]. It is regarded as a deep learning technique with the ability to differentiate between certain respiratory organ parenchyma image patches and non-lung parenchyma image patches. CNNs represent a significant advancement in image recognition. CNN is employed in the study of visual imagery [40,41].

The procedure of CNN is as follows:

- It begins with inserting an image.
- A function map is created by administering particular filters to it.
- To increase non-linearity, ReLU (Rectified Linear Unit) feature is used.
- Each feature map is carried out by a pooling layer.
- The pooled images are flattened into one prolonged vector.
- The vector is exerted into a completely related artificial neural network.
- The very last layer offers the “voting” of the classes. Processes the competencies via the community.
- The training is done via forwarding propagation and back-propagation for a certain period. The practice continues until a well-defined neural network with illustrated weights and function detectors is achieved.

The CT scans are segmented into 32 patches for segmentation, and each voxel is victimized for the primary aim of entering into the practiced CNN. Each patch is labeled with a 1 or 0, denoting lung parenchyma (LP) or non-lung parenchyma (NLP), at the same time (NLP). To reap the segmentation impacts of lung parenchyma, the hollow amid the platter volume is eventually packed [28]. Therefore, the early analysis for planning, monitoring, and remedy to establish the reference for longitudinal follow-ups can be carried out by an explicit CT-based AI system [29,42].

A COVID-19 diagnostic tool, the CT scan is used in China to observe affected people having fever and suspected contamination via CT lung scan as a medium of AI to examine infection in patients and require clinical imaging. Lesions within the lungs of NCP patients are perceptible from those resulting from bacteria. The chest CT scans can identify the difference. For example, shards of glass or reticular lines in the opaque lesions that look like abnormal paving tiles resemble the cloudy lesion motifs that occur across the peripheries of both lungs. Bacterial pneumonia lesions are generally concentrated in a single lung. They do not resemble shards of glass [20,43].

### 2.4. Significance of Statistical Analysis of AI in Healthcare

There are several studies presenting the statistical analysis of AI in healthcare. The significance of AI has been measured by perceived benefits with perceived risks using structural equation modeling [44]. The technological concern has occupied more weightage in perceived risk. Similarly, a survey report has emphasized three aspects of AI such as technological implementation, policy setting, and economic impact measurement by statistical survey [45]. As per the analysis of WHO, 70% countries of the globe have pointed out the lack of integration is the principal barrier to AI [46]. A real-time analysis of digital technologies in healthcare has been focused on early detection and diagnosis of diseases [47].

## 3. Methodology

### 3.1. Significance of ISM Methodology

ISM is a process for identifying and summarising linkages between several components that form a problem or issue. It is an interactive technique for building the interrelationships between the elements of a complicated system that incorporates expert input [48,49]. The advantages of ISM include that it requires fewer specialists than other methods such as the Delphi method and structural equation modelling, and it produces a structured model from unstructured and ambiguous raw data. ISM transforms an unstructured and ambiguous model into a well-defined, structured model [50]. It emphasises the components’ direct and indirect relationships and provides a clear picture of the problem [51]. It also emphasises both the long-term and short-term aspects of a problem [48]. ISM combines the opinions of professionals with their in-depth knowledge in the most efficient manner possible. As a result, this practical technique can provide a quick managerial perspective [52]. The interdependencies of the elements affecting the AI in healthcare sector during COVID-19 are highlighted in this study. As a result, ISM is an appropriate instrument for this task.

**Figure 3:**
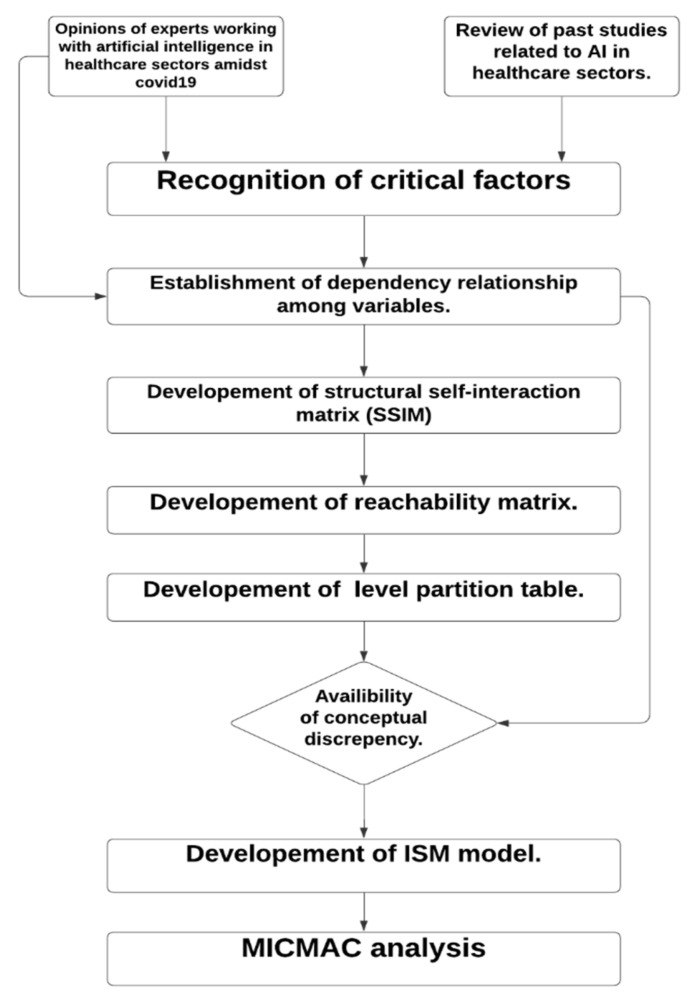
Flowchart of solution methodology.

In the first phase, we identified the critical factors affecting the diagnosis and prognosis of COVID-19; we reviewed the past literature and sought 20 expert opinions. The 20 experts included 15 medical doctors with at least 10 years of experience in a related field and working in the esteemed hospitals, and 5 experts who are working as chief medical officers in reputed companies that work in the field of medical image analysis using AI. Next, Interpretive Structural Modeling (ISM) was used to build a standard model of associations among critical factors, and MICMAC analysis was performed to categorize the factors. A total of 11 factors were taken which are responsible for the COVID-19 diagnosis and prognosis as shown in Table 1. The flowchart of the solution methodology is mentioned in Figure 3. Table 2 lists the ISM steps.

### 3.2. The ISM Methodology

Steps of ISM methodology are as follows:

Step 1: Development of a structural self-interaction matrix (SSIM);
Step 2: Construction of a reachability matrix;
Step 3: Level partitions;
Step 4: Classification of factors; and
Step 5: Formation of Interpretive Structural Modeling (ISM).

### 3.3. Development of Structural Self-Interaction Matrix (SSIM)

The relationship between different critical factors of COVID-19 used for the diagnosis are given by four symbols. This shows the direction of causality between the parameters x and y (in this case x y):

(1) V: x leads to y.
(2) A: y leads to x.
(3) X: x and y are related or lead to one another.
(4) O: the x and y parameters are independent.

The SSIM is constructed based on the relationship. SSIM is shown in Table 3.

### 3.4. Construction of Reachability Matrix

The structural self-interaction matrix (SSIM) was turned into a binary matrix termed reachability by inserting 1 and 0 for V, A, X, and O. The following rules govern the substitution of 1 and 0:

Rule 1: If the SSIM entry for (x, y) is V, the reachability matrix entry for (x, y) is 1 and the (y, x) entry is 0.
Rule 2: If the SSIM entry for (x, y) is A, the reachability matrix entry for (x, y) is 0 and the (y, x) entry is 1.
Rule 3: If the SSIM’s (x, y) entry is X, the reachability matrix’s (x, y) entry will be 1 as well, as will the (y, x) entry
Rule 4: If the SSIM’s (x, y) entry is O, the reachability matrix’s (x, y) entry will be 0 as well, as will the (y, x) entry.

The reachability matrix is shown in Table 4.

### 3.5. Carrying out Level Partitions

The reachability set and antecedent set for each factor are derived from the final reachability matrix. The reachability set contains other elements that may help achieve the element as well as the element itself, whereas the antecedent set includes other elements that may help achieve the element as well as the element itself. The intersection of these sets is then calculated for all elements. The top-level element in the hierarchy would not assist in achieving any element above their own, and the top-level element in the ISM hierarchy is the one with the same reachability and intersection sets. When the hierarchy’s top-level element is discovered, it is separated from other components, and the next level of elements is discovered using the same method. The digraph and final model are made by identified models. Level partitions are shown in Table 5.

### 3.6. Classification of Factors

On the basis of driving strength and dependability, the factors influencing the COVID-19 verdict are divided into four categories. These factors are relatively separated from the system, with which they have only a few weak links:

C1: Autonomous factors: low driving force and low reliance.
C2: Dependent aspects include a lack of driving power and a high level of reliance.
C3: Significant driving power and strong reliance are linkage factors.
C4: Self-contained factors with high driving power and little reliance.

**Figure 4:**
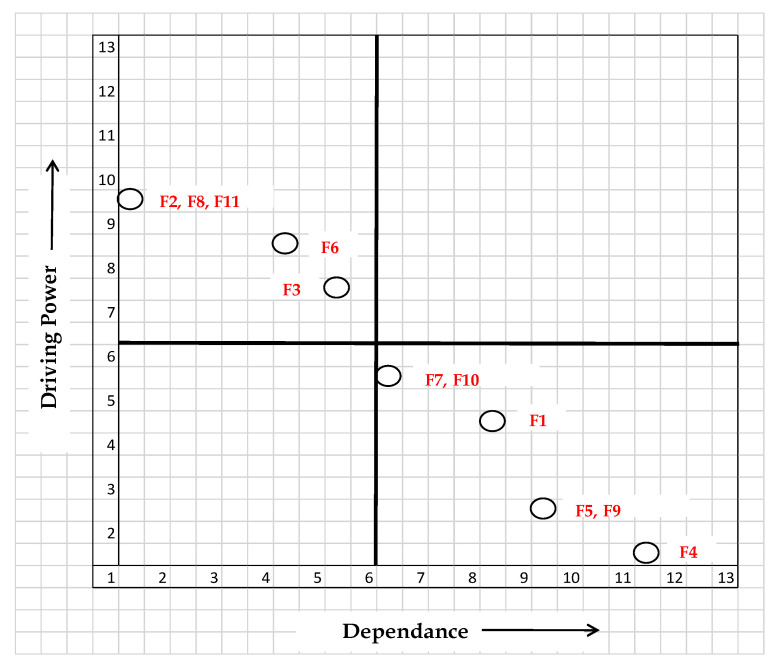
Driving power and dependence diagram.

Figure 4 shows the driving and dependence power of the critical factors

### 3.7. Formation of the Interpretive Structural Model

The ISM is constructed from the final reachability matrix’s vertices or nodes, as well as the lines of edges. An arrow pointing from x to y depicts the relationship between the variables x and y.

**Figure 5:**
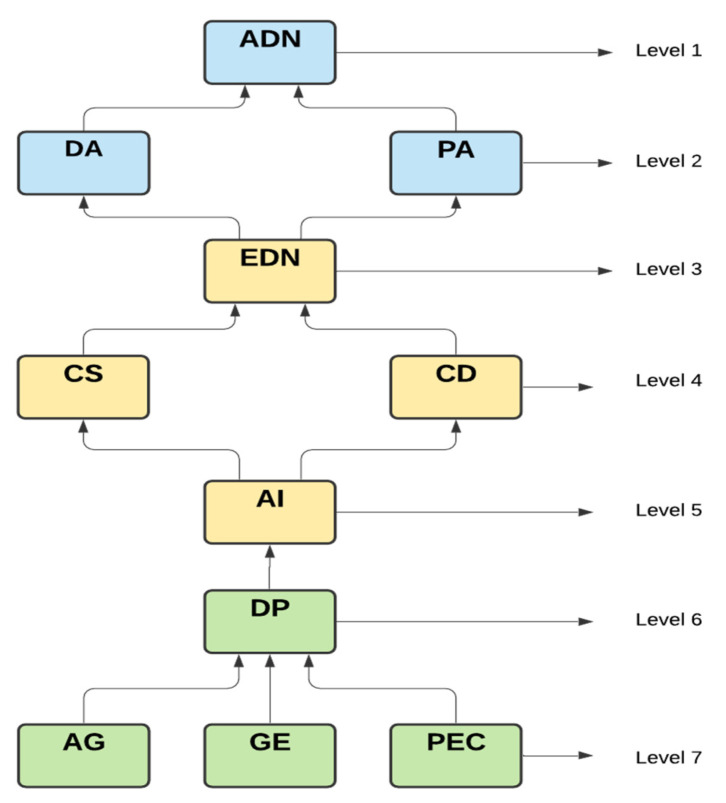
ISM Model.

A digraph or directed graph is the name given to this type of graph. The directed-graph is finally turned into ISM when the transitiveness is eliminated as shown in Figure 5.

## 4. Result and Discussion

There is not a single autonomous variable. These show up as the weak driver and weak dependent in most cases. Other system variables are unaffected by these variables. There are no autonomous factors discovered, and the system appears to be somewhat detached. There are only a few links in this, and none of the other variables are affected. Recognizing the factors that influence the early diagnosis of new diseases. We can observe from the digraph that there are no linking variables with a high driving force and a high degree of dependence. These variables are volatile because any changes they undergo will undoubtedly have an impact on others. As a result, there is no reason to believe that any of the variables included in this study are unstable. Independent variables are variables that have a high driving power and will assist companies in achieving their goals. Because the driving variables have a more strategic direction, performance can be improved by continuously enhancing them.

(1) There are no autonomous factors, and they appear as a weak driver and weak dependent. These variables have no effect on other variables and have only a few weak linkages.

(2) The contract data, combined data, early detection of NCP, diagnosis analysis, prognosis analysis, and accurate detection are the six factors categorized under weak drivers and strongly dependent, called the dependent category. They are the highest-ranking members of the ISM. These factors, which are categorized as dependent factors, constitute the goal of any company.

(3) Linkage variables have a high driving force as well as a high degree of reliance. These variables are volatile because any changes they undergo will undoubtedly have an impact on others. As a result, there is no reason to assume that any of the variables used in this study are volatile.

(4) Age, pre-existing conditions, gender, digital phenotyping, and AI are five elements that are grouped together as drivers. These components are classified as independent variables since they are at the bottom of the model and have a strong driving force that helps companies achieve their goals. Because the driving variables have a more strategic direction, performance can be improved by continuously enhancing them.

The result shows that the short-term factors i.e., age, sex, and pre-existing condition are the key factors and has a hold on more driving power for other factors to achieve the the long-term goal i.e., accurate detection of NCP.

The analysis of ISM result illustrates that early detection of NCP (EDN) depends on combined speed (CS) with contract data (CD) which is governed by AI and validates the outcome of past researcher’s work [44,47]. Similarly, the technological implementation through diagnosis and prognosis analysis helps to achieve the accurate detection of NCP (ADN) and it supports the previous research [45]. The result of ISM points out that a lack of integration among AI, CD, and CS results in a lack of EDN to achieve ADN in the long term, which confirms the analysis derived previously [46].

## 5. Managerial Implications

Regulators, governments, healthcare industry executives, and consumers will be able to understand the essential aspects that affect AI in the healthcare sector as a result of our research. Managers and decision-makers should concentrate on the ISM model’s inputs and outputs. A literature review and expert opinions were used to form the inputs. The outputs identify interdependencies as well as the importance of various aspects in the short and long run. In numerous industries, the model will be deployed and assessed in a cross-sectional approach. The outcomes will stimulate managers’ curiosity, and they should plan the resources necessary for successful execution. Managers must conduct workshops and training on AI and its benefits to their employees. Existing educational institutions and specific training schools may be involved. Managers must be vigilant when sharing knowledge to avoid losing a competitive advantage. With technological advancements, there is a significant possibility for using this data to analyze healthcare. Knowledge of technical aspects will be useful during organizational preparation. All firms must now implement cutting-edge AI technologies [7,53]. Smart contracts, privacy, and data security are among its essential features, and switching to new (better) future platforms is simple. Existing open-source platforms are prohibitively expensive when used as proprietary infrastructure. AI technology should be implemented immediately, as early adoption of the technology will give us a massive head start. The use of AI detects the presence of patterns and correlations, resulting in predictive analytics for enhancing healthcare delivery [8,18,44].

## 6. Practical Implications

To secure the privacy of healthcare data, healthcare decision-makers must use AI. This level of anonymity facilitates the use of AI and federated learning, which improves organizational efficiency. In the era of coronavirus disease in 2019, AI can better predict infection and is applicable globally. As data digitization advances, so does the demand for privacy, as well as the desire for societal improvement. The requisite systems can be built on edge of AI technology. Researchers, developers, scientists, healthcare experts, and policymakers must strike a balance between the need to innovate and measures to ensure that AI’s societal and economic benefits are widely distributed. The innovations emerging from AI will radically impact society for the better in the next decades if humanity embraces AI with a more inquisitive mind [27,45,46].

## 7. Conclusions and Future Work

COVID-19 diagnostic and prognostic models are accessible and seem to show incredible top-notch performance. These models provide: (1) improved remedy consistency and selection making by developing useful algorithms; (2) proper treatment regimens; (3) the destiny direction of the disease and its reappearance; (4) digital approaches used to provide treatment at an early stage. Additional diagnostic checks are going to be useful to ultimately halt the pandemic, restrict the economic damage from lockdowns, and avoid a rebound as soon as the restriction is relaxed. The whole situation makes a case for extensive diagnostic testing of the populace to permit people to come back to figure out situations that they may no longer be infectious. They conjointly require extra arbitrarily sampled tests to boost our estimates of the share of the population with the virus that lives well.

The value of AI comes into play with the aid of decreasing the weight of clinicians in a situation along with the current COVID-19 trends. Therefore, these AI models can serve as worthwhile yet accessible parameters to combat COVID-19 diagnosis and prognosis. The consequences display that the accurate detection of NCP (ADN) is being monitored, initially through the short-term factors, i.e., age, gender, and pre-existing conditions. The primarily hierarchy- based ISM further defines both the long-time period and short-time period factors. In the future, the research can be extended with more factors to find more clarity in the relationship, and the study of latent variables will add causal relationship among them.

Furthermore, the proposed ISM version serves as a good guiding principle for improving the overall performance of the diagnosis and prognosis of COVID-19 with the integration of AI. The paper provides an interpretive structural model to widen the skeleton of the complex interactions and significance amongst identified critical elements.

## Figures and Tables

**Table 1:** Factors responsible for the COVID-19 diagnosis and Prognosis.

Factors	Name	Notation
F1	Early detection of NCP	EDN
F2	Age	AG
F3	Artificial Intelligence	AI
F4	Accurate detection of NCP	ADN
F5	Diagnosis analysis	DA
F6	Diagnosis phenotyping	DP
F7	Contract data	CD
F8	Pre-existing condition	PEC
F9	Prognosis Analysis	PA
F10	Combine spend	CS
F11	Gender	GE

**Table 2:** ISM steps.

Sl. No	STEPS	FOCUS
1	Establishment of a structural self-interaction matrix (SSIM)	Define pairwise relationships among identified critical dimensions of AI in healthcare sector
2	Create a reachability matrix	Determining driving & dependent powers
3	Level Partitioning	Define structural levels (factor level partitioning)
4	ISM Modelling	Develop an ISM model using reachability matrix & level partitioning
5	MICMAC analysis	Classify critical dimensions of AI in healthcare sector into four categories (drivers, dependence, autonomous factors, and linked factors) via MICMAC analysis.

**Table 3:** Structural self-interaction matrix.

	EDN	AG	AI	ADN	DA	DP	CD	PEC	PA	CS	GE
EDN		A	A	V	V	A	A	A	V	A	A
AG			V	V	V	V	V	O	V	V	O
AI				V	V	A	V	V	V	V	A
ADN					A	A	A	V	A	A	A
DA						A	A	V	O	A	A
DP							V	V	V	V	A
CD								V	V	O	A
PEC									V	V	O
PA										A	A
CS											A
GE											

**Table 4:** Reachability matrix.

F. No	F1	F2	F3	F4	F5	F6	F7	F8	F9	F10	F11	Driving
F1	1	0	0	1	1	0	0	0	1	0	0	4
F2	1	1	1	1	1	1	1	0	1	1	0	9
F3	1	0	1	1	1	0	1	0	1	1	0	7
F4	0	0	0	1	0	0	0	0	0	0	0	1
F5	0	0	0	1	1	0	0	0	0	0	0	2
F6	1	0	1	1	1	1	1	0	1	1	0	8
F7	1	0	0	1	1	0	1	0	1	0	0	5
F8	1	0	1	1	1	1	1	1	1	1	0	9
F9	0	0	0	1	0	0	0	0	1	0	0	2
F10	1	0	0	1	1	0	0	0	1	1	0	5
F11	1	0	1	1	1	1	1	0	1	1	1	9
Dependance	8	1	5	11	9	4	6	1	9	6	1	

**Table 5:** Carrying out level partitions.

Factor No	Reachability Set	Antecedent Set	Intersection Set	Level
1	1,4,5,9	1,2,3,6,7,8,10,11	1	Third
2	1,2,3,4,5,6,7,9,10	2	2	Seventh
3	1,3,4,5,7,9,10	2,3,6,8,11	3	Fifth
4	4	1,2,3,4,5,6,7,8,9,10,11	4	First
5	4,5	1,2,3,5,6,7,8,10,11	5	Second
6	1,3,4,5,6,7,9,10	2,6,8,11	6	Sixth
7	1,4,5,7,9	2,3,6,7,8,11	7	Fourth
8	1,3,4,5,6,7,8,9,10	8	8	Seventh
9	4,9	1,2,3,6,7,8,9,10,11	9	Second
10	1,4,5,9,10	2,3,6,8,10,11	10	Fourth
11	1,3,4,5,6,7,9,10,11	11	11	Seventh
